# Skin cancer screening practices of UK hairdressers and barbers for their customers: a preliminary study

**DOI:** 10.1093/skinhd/vzaf015

**Published:** 2025-04-22

**Authors:** Helen Fleming, Chris Wells, Andrew Williams, Rebecca Stores

**Affiliations:** School of Dental, Health & Care Professions Faculty of Science and Health, University of Portsmouth, Portsmouth, UK; School of Dental, Health & Care Professions Faculty of Science and Health, University of Portsmouth, Portsmouth, UK; School of Health & Rehabilitation Science, Health Sciences University, Bournemouth, UK; School of Dental, Health & Care Professions Faculty of Science and Health, University of Portsmouth, Portsmouth, UK

## Abstract

**Background:**

Head-and-neck skin cancers have a worse prognosis than those that develop elsewhere on the body. Self-screening this area for suspicious skin changes can be difficult. Hairdressers and barbers observe this area closely during hair appointments and could bring their customers’ attention to suspicious skin changes earlier.

**Objectives:**

To investigate a sample of UK hairdressers’ and barbers’ skin cancer education, customer screening practices and influences on screening, and to compare hairdressers’ and barbers’ screening practices.

**Methods:**

Stratified random sampling was utilized to select hairdressers and barbers working in a UK city. Participants were invited to complete a survey.

**Results:**

Thirty-seven participants completed the survey. Five per cent reported having had skin cancer awareness training and 24% were screening customers. Thirty-five per cent had advised a customer of a suspicious mole or skin lesion; of these participants, 39% had had customers diagnosed with skin cancer. ‘Not having received training’ was reported by 65% of participants as a deterrent to screening. Knowing someone who had experienced skin cancer was significantly associated with screening and advising customers of suspicious skin changes. Most participants (92%) indicated they would like, or maybe like, skin cancer awareness training.

**Conclusions:**

In this UK city study, perceived knowledge of the signs and symptoms of skin cancer appeared to arise from knowing someone who had experienced skin cancer rather than formal training. Lack of skin cancer education was a deterrent to screening, but most participants would like training. Trained hairdressers and barbers could potentially provide regular head-and-neck skin screening for customers.

## Teaser

Regular self-screening for suspicious moles or skin lesions can help identify early skin cancers, improving the prognosis of the patient; however, most skin cancers develop in the difficult to self-screen head-and-neck area. Hairdressers and barbers observe the head-and-neck area closely during routine hair appointments and could advise customers of suspicious skin changes. UK hairdressers’ and barbers’ skin cancer screening practices for their customers have not previously been investigated. The study aimed to determine whether a stratified random sample of UK hair professionals had received training in skin cancer awareness, and were screening or advising their customers, to investigate influences and deterrents to screening and advising, and attitudes towards skin cancer awareness training.

**What is already known about this topic?**

- The limited number of previous studies conducted have reported that few hair professionals have received education in skin cancer awareness; however, some screen their customers for skin cancer.
- The most common reasons reported for not screening customers were lack of training and confidence in detecting skin cancers.
- Most hair professionals were interested and willing to learn more about skin cancer and screen customers with proper training.

**What does this study add?**

- UK hair professionals’ skin cancer screening practices for customers have not previously been researched.
- Few (5%) participants had received skin cancer awareness education; however, 24% were screening and 35% had advised customers.
- Of participants who had advised a customer, 39% had a customer diagnosed with skin cancer.
- Screening and advising customers were significantly associated with knowing someone who had experienced skin cancer.
- Most participants would like skin cancer awareness education.

Skin cancer is the UK’s most common cancer.^1^ Since the 1990s, the incidence of melanoma and nonmelanoma skin cancer (NMSC) have risen by 147%^2^ and 169%,^3^ respectively. Over 224 000 cases of skin cancer were diagnosed in England in 2019,^4^ costing the National Health Service (NHS) an estimated £150.5 million.^5^

NMSCs constitute 90% of all skin cancers diagnosed in the UK, causing approximately 720 deaths per year.^3^ Most (80%) develop in the head-and-neck area due to frequent sun exposure.^6^ NMSCs can cause significant cosmetic disfigurement,^7^ resulting in lasting psychological impacts for some patients.^8^

Head-and-neck melanomas have a poorer prognosis than those developing elsewhere on the body.^9^ Difficulty in self-screening the head-and-neck area may cause delay in the discovery of suspicious skin changes.^10^

Detection of suspicious moles or skin lesions relies on self-surveillance or surveillance by others, presuming those screening possess awareness of the signs of skin cancer. However, a survey of 3600 UK residents found that only 23% of participants could identify the most common signs of NMSCs, and 69% did not recognize NMSC as a form of skin cancer.^11^ Similarly, a UK study of the experiences of 76 patients with NMSC found that knowledge of the signs and symptoms of NMSC was poor, both prediagnosis and post-treatment. Hairdressers had alerted 13% of clients to their NMSCs and encouraged them to seek a medical opinion.^12^

Hairdressers and barbers hold a unique position, observing the head-and-neck area under bright lights during routine hair appointments, allowing them to closely scrutinize this area and notice suspicious skin changes. In this difficult-to-self-screen area, alerting customers to suspicious moles or lesions could potentially improve the earlier detection of skin cancer.

Few studies have investigated this topic; however, previous studies conducted in the USA found low levels of education in skin cancer awareness in hairdressers and barbers, although some were screening and advising customers. Bailey *et al*.^10^ surveyed 203 Texan hair professionals on their frequency of screening customers’ scalp, neck and face for suspicious lesions. Most (72%) had no skin cancer training and poor knowledge of the ABCDE (asymmetry, borders, colour, diameter, evolving)^13^ melanoma criteria. However, 37% were screening 50% of their customers’ scalps, 29% screened 50% of customers’ necks and 58% had recommended a customer seek further investigation for a suspicious mole. Almost half (49%) indicated receptiveness to undertaking skin cancer awareness training.

Similarly, a study of 108 Californian hairdressers’ scalp-and-neck melanoma detection and referral practices^14^ found that over 50% had not received skin cancer training, 85% were unfamiliar with the ABCDE melanoma detection criteria, and 27% rarely or never examined customers’ scalps. However, 69% had referred customers to dermatologists for suspicious lesions, with 36% of referrals leading to a medical diagnosis. Additionally, 92% of the hairdressers expressed interest in learning more about skin cancer detection.

A study reviewing 6 years of scalp-and-neck melanoma referrals at an oncology centre revealed additional benefits for customers whose undiagnosed skin cancer was noticed by their hairdresser.^15^ Of the 128 patients with melanoma reviewed, 68 had detected their melanoma themselves, 28 were unspecified, 15 were detected through physicians, 12 through hairdressers, 5 through family/friends and 4 through a spouse. Significantly, the study found that patients whose melanomas were detected by their hairdresser presented 13 years younger than average and at an earlier stage than patients whose melanomas were detected through other sources, including physicians. The study concluded that hairdressers and barbers could aid the earlier detection of aggressive head-and-neck melanomas. The advantages of hairdressers having skin cancer knowledge were also noted in a small study,^16^ which highlighted three cases in which hairdressers had detected customers’ scalp melanomas and were persistent in encouraging them to seek medical attention.

The studies discussed show that hair professionals have previously been identified as a potential head-and-neck screening resource. Utilizing UK hair professionals to screen their customers for suspicious lesions has also previously been considered. Articles promoting skin cancer awareness in UK professional hairdressing magazines^17^ were discovered from 2012; the UK’s National Hair and Beauty Federation has published advice for its members on how to spot the signs of skin cancer;^18^ the Karen Clifford Skin Cancer charity (Skcin) developed an accredited skin cancer awareness programme for hair professionals;^19^ and the Vocational Training Charity Trust provides a skin cancer awareness course aimed at non-healthcare professionals, including hair professionals.^20^ However, a systematic literature review conducted by the first author discovered no studies establishing UK hairdressers’ and barbers’ skin cancer education or screening practices. To address this gap in knowledge, the authors conducted a preliminary study via a survey that aimed to establish the current skin cancer education and screening practices of UK hairdressers and barbers.

## Materials and methods

The primary objective was to determine the number of participants who had received skin cancer awareness training and were screening and advising customers.

Secondary objectives were to analyse influences and deterrents to screening, willingness to screen and receive training, and to compare screening practices between hairdressers and barbers to establish commonalities or variations.

A stratified random sampling method was used to recruit hairdressers and barbers to complete a survey. The researcher selected the city of Portsmouth, UK, to conduct the study as Portsmouth melanoma incidence per 100 000 persons recorded between 2017 and 2019 was 38.1^21^ vs. 28.2 overall in England.^2^ Participants were eligible to complete the survey if they worked in the city of Portsmouth as a hairdresser or barber within one of the randomly selected hair salons or barber shops.

The authors identified hair salons and barber shops within the city using Google Maps. Two hair salons and two barbershops located in each PO1–PO6 postcode area, totalling 24 establishments, were required in order to generate a socio-demographically mixed representative sample of the city. The authors visited hairdressers and barbers working in these establishments face-to-face and invited them to complete a paper survey. The authors collected the completed surveys 1 week later.

The 22-question paper survey included a QR link to an identical online survey. Questions were based on previous hairdressers’ skin cancer screening practices studies.^10,14^ The survey investigated participants’ skin cancer awareness education, screening practices, confidence to screen, deterrents to screening and willingness to learn about skin cancer. Two hairdressers and two barbers purposefully chosen outside of the study area piloted the survey to review survey clarity, readability, relevance and an acceptable timeframe for completion.

Data were analysed using SPSS 28 software (IBM, Armonk, NY, USA). Descriptive statistics were used to describe participants’ demographics. An independent samples *t*-test was used to test for any significant difference in age between those screening for skin cancer and those that did not, and for years in occupation between those that advised on suspicious moles or lesions and those that did not. Pearsons χ^2^ statistics were used to investigate potential associations between screening and advising customers and knowing someone with skin cancer. An independent samples *t*-test was performed to compare hairdressers’ and barbers’ screening practices. Participants’ open-ended question responses supplemented the quantitative data.

## Results

Questionnaires were completed by 37 participants from 24 establishments, equally representing the city’s postcode areas; 31 were completed on paper and 6 online.

Table 1 shows the demographic characteristics of participants and participant groups. Participants were 65% women and comprised 17 hairdressers (46%), 17 barbers (46%) and 3 identifying as hairdressers/barbers (8%). The mean (SD) age of the participants was 36.89 (12.14) years and their mean (SD) number of years in the occupation was 17.00 (11.23).

**Table 1:** General characteristics and demographics of participants (*n* = 37)

Occupation	No. of participants (*n*)	Gender		Mean age, years (SD)	Mean occupation, years (SD)
Hairdresser	17 (46%)	Female, 15 (88%)	Male, 2 (12%)	32.88 (11.69)	15.59 (11.23)
Barber	17 (46%)	Female, 6 (35%)	Male, 11 (65%)	40.41 (10.13)	17.24 (13.93)
Hairdresser/barber	3 (8%)	Female, 3 (100%)	Male, 0	39.67 (22.03)	23.67 (22.03)
Total	37	Female, 24 (65%)	Male, 13 (35%)	36.89 (12.14)	17.00 (11.23)

Table 2 shows participants’ responses to skin cancer awareness training, screening and advising practices, and personal experience of skin cancer. Five per cent reported they had received skin cancer awareness training at college, and 24% reported screening customers, of whom 100% were screening customers’ scalps, 56% were screening necks, 44% were screening ears and 33% were screening customers’ faces. Primary reasons for participants not screening customers were ‘I have not had training to screen for suspicious moles or skin lesions’ (65%) and ‘I am not confident to screen for suspicious moles or skin lesions’ (24%). Thirty-five per cent reported advising a customer of a suspicious mole or skin lesion. Of these, 39% reported a customer receiving a diagnosis of skin cancer. Participants’ personal experiences of skin cancer showed that 3% had personally experienced skin cancer and 54% knew someone who had had skin cancer.

**Table 2:** Responses to hairdressers’ and barbers’ current skin cancer awareness training, screening and advising practices, and personal experience of skin cancer

Question	Total (*n* = 37)
Have you previously had skin cancer awareness training?	
No	34 (92)
Yes	2 (5)
Don’t know	1 (3)
Are you screening your customers for suspicious moles/skin lesions?	
No	28 (76)
Yes/sometimes	9 (24)
If yes, what areas of your customers do you screen?	
Scalp	9 (100)
Neck	5 (56)
Ears	4 (44)
Face	3 (33)
Other	2 (22)
What is the main reason that you would not screen customers for suspicious moles/skin lesions?	
I have not had training to screen for suspicious moles/skin	24 (65)
I am not confident to detect suspicious moles/skin lesions	9 (24)
It is not part of my job	6 (16)
I am not confident to discuss suspicious moles/skin lesions with customers	2 (5)
I don’t have time to screen	1 (3)
Have you advised a customer to seek medical advice for suspicious moles/skin lesions?	
No	24 (65)
Yes	13 (35)
If yes, did the customer receive a medical diagnosis of skin cancer?	
No	6 (46)
Yes	5 (39)
Don’t know	2 (15)
Have you previously been diagnosed with skin cancer?	
No	36 (97)
Yes	1 (3)
Do you know someone who has previously been diagnosed with skin cancer?	
Yes	20 (54)
No	16 (43)
Don’t know	1 (3)

Data are presented as *n* (%).

An independent *t*-test showed no statistically significant difference in age between those that screened and those that did not [t = 0.564, degrees of freedom (df) = 10.2, *P* = 0.59, two tailed] and no significant difference in years in occupation between those who screened and those who did not (t = 0.918 df = 9.825, *P* = 0.38, two tailed). There was no significant difference in age between those who advised and those who did not (t = 1.240 df = 35, *P* = 0.22, two tailed) and no significant difference in years in occupation between those who advised and those who did not (t = 1.951 df = 35, *P* = 0.06, two tailed), although this approached the 0.05 significance level. The Pearsons χ^2^ test to determine associations between screening and advising customers and knowing someone who had experienced skin cancer found a statistically significant association between screening and advising customers of suspicious moles or skin lesions and knowing someone with skin cancer. Some 89% of participants screening customers knew someone who had been diagnosed with skin cancer (χ^2^ = 5.40, df = 1, *P* < 0.02), and 85% of those advising customers knew someone who had been diagnosed with skin cancer (χ^2^ = 6.96, df = 1, *P* < 0.008).

**Figure 1:**
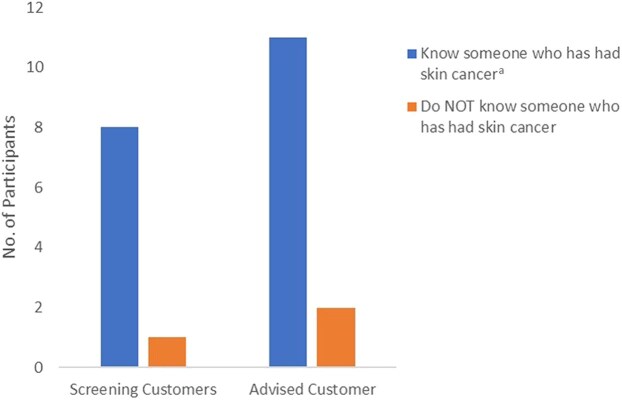
Number of participants screening and advising customers who know someone who has had skin cancer. ^a^The bar chart shows that 89% of participants screening customers knew someone who had been diagnosed with skin cancer [χ^2^ = 5.40, degrees of freedom (df) = 1, *P* < 0.02], and 85% of those advising customers knew someone who had been diagnosed with skin cancer (χ^2^ = 6.96, df = 1, *P* < 0.008).

Of those participants who responded, 54% knew someone who had had skin cancer; however, Figure 1 shows the number of participants screening and advising customers who knew someone who had been diagnosed with skin cancer was much higher.

Participants’ responses to the open-ended questions, shown in Table 3, provide supplementary information and add support and richness to the quantitative data.

**Table 3:** Participant comments on advising customers, and attitudes toward training

**How did customer react to being advised to seek medical advice?**
- Happened a few times, usually they respond well and say they will contact the doctor
- Sometimes they feel embarrassed I assume but I always tell them we recognise these things
- She was happy/grateful that I informed her
- Fine, it was an area the customer could not see for themselves
- Thankful for the concern and heads up
- He was quite unsettled as he already has moles on his head. As a result he went and got them checked
- They didn’t know they had it. I explained and showed her in the mirror and she managed to get it looked at and sorted and was very grateful that I showed her because she didn’t know
- Most were grateful
- With concern and gratitude
- Customer pointed out that they had already noticed change in mole – attended physician of own accord
- Some follow it up, some don’t. When they come back in and say they haven’t been then you nag them again
**Do you believe skin cancer awareness training should be taught as a standard part of hairdresser or barber courses?**
- It should be on the course as part of the qualifications
- Would be a great help to saving more people
- Nowadays people can get easily offended when you advise them about any skin changes therefore I think it’s better to leave it to people responsible to do so (e.g. GP and doctors) because they have full duties and responsibilities
- Would depend on level of training
- It should be offered
- Hairdressers/barbers/beauticians/nail/tattooists should already look for other health conditions, contraindications to treatments and I feel there could be more education on skin cancer awareness
- I think it would be wise if we were able to recommend to a doctor to seek further help
- Although I do see/speak to customers with skin concerns I don’t have the knowledge and confidence to give any advice, I’d advise them to contact their doctor
**Would you like to receive skin cancer awareness training/further training?**
- I would like to see it added to the training but unfortunately I think it won’t happen, if a free course was offered and I wasn’t working I would attend
- Definitely
- I would not like to notify my customers if they had cancer but believe it would be valuable knowledge to have just in case
- More education on skin cancer is needed for everyone
- Hairdressing is about hair not medical for people
- It would be a great addition
- This training would be great as it would give us confidence to raise concerns with customers and also answer questions they might have
**What would deter you from undertaking skin cancer awareness training?**
- Medical is a different industry
- Cost
- Not medical trained to give appropriate advice and I don’t want to miss diagnose
- Time, work is busy

GP, general practitioner.

One response echoed the findings of Pillemer *et al*.^16^ regarding the persistence of hairdressers and barbers in encouraging customers to seek a medical opinion for suspicious moles.

‘Some follow it up, some don’t. When they come back in and say they haven’t been then you nag them again.’

Responses revealed that the reaction of most customers who were advised by their hairdresser or barber of a suspicious mole or skin lesion was positive.

Participants’ responses showing attitudes toward advising customers and attitudes toward skin cancer awareness training are shown in Table 4. Fifty-eight per cent of participants reported they believed it should, or sometimes should (22%), be the role of a hairdresser or barber to advise their customer of suspicious skin changes. Almost three-quarters (74%) reported that hairdressers or barbers should have skin cancer awareness included in their training. Over half (67%) indicated they would like skin cancer awareness training; 25% indicated they would maybe like training. Participants open responses identified, ‘cost,’ ‘time’ and concern over ‘misdiagnosis’ as deterrents to undertaking training.

**Table 4:** Participant survey responses showing attitudes to advising customers, and attitudes to skin cancer awareness training

Question	Total (*n* = 37)
Should it be part of a hairdresser’s/barber’s role to advise customers of suspicious skin changes?	
Yes	21 (57)
Sometimes	8 (22)
No	4 (11)
Don’t know	3 (8)
Do you believe skin cancer awareness training should be taught as a standard part of hairdresser or barber courses?	
Yes	26 (74)
Don’t know	6 (17)
No	3 (9)
Would you like to receive skin cancer awareness training/further training?	
Yes	24 (67)
Maybe	9 (25)
No	3 (8)

Not all questions were answered by all respondents. Data are presented as *n* (%).

**Figure 2:**
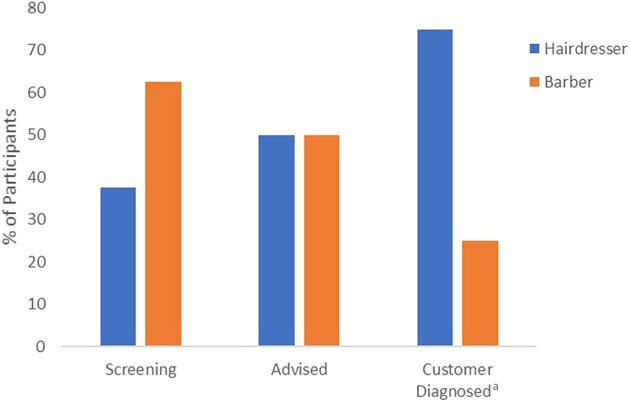
Comparison of the screening of customers by hairdressers and barbers, advising customers and those who had advised customers whose customers had gone on to receive a diagnosis of skin cancer. ^a^A statistically significant difference was observed between the groups for those who had had a customer diagnosed with skin cancer t = 0.73, degrees of freedom = 10, *P* < 0.05, 95% confidence interval −1.36 to 0.69.

To perform a direct comparison of hairdressers, and barbers, screening practices, the results of participants who identified as hairdressers/barbers were excluded. Figure 2 shows the comparison analysis results: 25% more barbers (63%) than hairdressers (38%) were screening customers; 50% of customers who were advised of suspicious moles or skin lesions were advised by hairdressers and 50% by barbers. Of the 39% of customers who were advised of suspicious moles or skin lesions and were known to have subsequently received a diagnosis of skin cancer, barbers detected 25% and hairdressers detected 75% – three times more. More barbers knew someone who had been diagnosed with skin cancer (58%) than hairdressers (42%). Of those hairdressers and barbers who indicated they would like, or maybe like, to learn more about skin cancer awareness, 50% were hairdressers and 50% were barbers. The results of the independent samples *t*-test found that although more barbers than hairdressers were screening customers, the differences between groups’ screening practices were not statistically significant [t = 0.79, df = 32, *P* = 0.12, 95% confidence interval (CI) −0.42 to 0.18]. There was also no statistically significant difference between the two groups in advising customers (t = 0.00, df = 32, *P* = 1.00, 95% CI −0.34 to 0.34). A statistically significant difference was observed between the groups for those who had had a customer diagnosed with skin cancer (t = 0.73, df = 10, *P* < 0.05, 95% CI −1.36 to 0.69).

## Discussion

This UK city study revealed that few hairdressers and barbers have received skin cancer awareness training. However, almost a quarter reported screening customers and over a third advising a customer of suspicious moles or skin lesions, with some customers later diagnosed with skin cancer. Deterrents to screening were identified as lack of training and confidence to screen. Knowing someone who had had skin cancer demonstrated a statistically significant association with screening and advising customers. Most participants indicated they would like to learn more about skin cancer awareness.

More barbers than hairdressers screen customers. However, the numbers advising customers were the same, while more hairdressers than barbers had advised a customer who had subsequently been diagnosed with skin cancer.

In comparison with previous study findings, Bailey *et al*.^10^ found that 18% of hair professionals had received skin cancer training, 37% were screening customers and 58% had advised a customer to seek medical advice. Roosta *et al*.^14^ discovered that 50% had received training, 70% were screening and 69% had advised customers to seek medical advice. The results of this UK study found significantly lower numbers of hairdressers and barbers with skin cancer training (5%), screening customers (24%) or advising customers to seek a medical opinion (35%).

The question arises regarding where those hairdressers and barbers who screen and advise customers of skin cancer signs acquired their knowledge. The statistically significant link between knowing someone who has had skin cancer and screening and advising customers may provide a credible answer. Bailey *et al*.^10^ also found that hair professionals who had experienced skin cancer personally or via an acquaintance’s diagnosis were more likely to examine customers’ skin. These findings may explain the disparity between skin cancer education and the number of hair professionals screening and advising customers, suggesting that increased awareness through a personal or family/friend’s experience of skin cancer may compensate for lack of training. The high number of hairdressers and barbers screening and advising customers who knew someone who had experienced skin cancer in this city with historically high rates of melanoma is notable.

An important finding was that, of those who had advised a customer of a suspicious mole or skin lesion, 39% led to a customer receiving a diagnosis of skin cancer. This compares favourably with the study findings of Roosta *et al*. (36%).^14^ These results demonstrate that participants were bringing customers’ attention to skin cancers that were undetected or perceived as benign, aiding them in earlier diagnosis and treatment.

The reasons participants reported for not screening were lack of training (65%) and lack of confidence to screen for suspicious moles or skin lesions (24%). These numbers correlate with the numbers who reported that they would like, or maybe like, to learn more about skin cancer (92%) and support previous study findings.^10,14^ These results suggest that hairdressers and barbers would be willing to learn more and screen their customers with proper training.

These survey findings are significant as studies have shown it cannot be assumed that those self-screening have an awareness of the signs of skin cancer.^11,12^ Hairdressers and barbers who routinely screen their customers head-and-neck area could provide some reassurance, particularly for those who have previously had skin cancer and are more likely to develop further skin cancers.^22^

Whether barbers are as likely to screen customers as hairdressers is also of significance. Men are more likely to develop skin cancer than women,^2^ and most barbers have predominantly male customers. It is therefore important to establish commonalities and variations between hairdressers’ and barbers’ screening practices. Interestingly, more barbers (63%) than hairdressers (38%) reported screening customers; this could be related to barbers’ customers generally having shorter or thinning hair, making the scalp easier to observe; however, the numbers advising customers of suspicious skin changes were equal. Of those who had advised customers, leading to a diagnosis of skin cancer, there was a variation between barbers (25%) and hairdressers (75%). However, this may not be significant due to the small number of customers it relates to.

The number of participants who responded that they would like, or maybe like, training in skin cancer awareness shows their willingness to learn more. The majority agreed it should be taught as a standard part of apprenticeship training. Although resources exist that could address this gap in knowledge,^18–20^ questions arise over awareness of these resources and potential deterrents to undertaking skin cancer education. Cost, time and concerns over misdiagnosis were reasons cited by participants when asked what might deter them from completing a skin cancer awareness course.

Training hairdressers and barbers to identify suspicious moles or lesions may also have negative consequences. While they can alert customers to seek further investigation, their role is not diagnostic. This could cause unnecessary distress if concerns turn out to be unfounded. Additionally, it may lead to increased general practitioner visits and dermatology referrals. These factors must be considered alongside the potential benefits of early skin cancer detection.

This study, conducted in a single city, may not represent the screening practices of hairdressers and barbers across the UK. Further research is needed to assess the nationwide training and interest in skin cancer awareness among UK hair professionals. No research currently compares the screening practices of barbers and hairdressers, so future studies with larger samples could determine whether both professionals detect suspicious moles or lesions equally.

Further research is required to explore the potential efficacy of training hairdressers and barbers in skin cancer awareness and whether this could increase earlier detection of head-and-neck skin cancers.

The results of this preliminary study indicate that hairdressers and barbers lack training and confidence to screen their customers but would be willing to learn more. Some are screening and advising their customers despite no formal training, appearing to rely on awareness gained through knowing someone who has had skin cancer. Providing training in skin cancer awareness for hairdressers and barbers could enable them to provide an extensive screening resource for their customers, inclusive of all ethnicities and socioeconomic demographics.

Further research in this area could facilitate the inclusion of skin cancer awareness training for apprentice UK hairdressers and barbers, and free training for established hairdressers and barbers. This could allow willing hairdressers and barbers to provide a nationwide head-and-neck screening resource for their customers, potentially aiding the earlier detection of skin cancer, improving patient prognosis and cutting NHS treatment costs.

## Data Availability

The data underlying this article will be shared on reasonable request to the corresponding author.
